# Molecular detection of the natural infection by trypanosomatid parasites in *Didelphis marsupialis* from a rural area in northern Colombia

**DOI:** 10.17843/rpmesp.2023.401.11573

**Published:** 2023-03-24

**Authors:** Marlon M. Ardila, Yoselin Villadiego, Leidi Herrera, Wendy Zabala-Monterroza, Alveiro Pérez-Doria

**Affiliations:** 1 Faculty of Basic and Biomedical Sciences, Universidad Simón Bolívar, Barranquilla, Colombia. Universidad Simón Bolívar Faculty of Basic and Biomedical Sciences Universidad Simón Bolívar Barranquilla Colombia; 2 Department of Animal Science, Faculty of Veterinary Science, Universidad de Concepción, Chillán, Chile. Universidad de Concepción Department of Animal Science Faculty of Veterinary Science, Universidad de Concepción Chile; 3 Interdisciplinary Group in Marine and Environmental Sciences (GICMARA), Basic Sciences Faculty, Universidad del Atlántico, Puerto Colombia, Colombia. Universidad del Atlántico Interdisciplinary Group in Marine and Environmental Sciences (GICMARA) Basic Sciences Faculty Universidad del Atlántico Puerto Colombia Colombia; 4 Center for Ecology and Evolution, Institute of Zoology and Tropical Ecology (IZET), Faculty of Sciences, Universidad Central de Venezuela (UCV), Caracas, Venezuela. Universidad Central de Venezuela Center for Ecology and Evolution, Institute of Zoology and Tropical Ecology (IZET) Faculty of Sciences Universidad Central de Venezuela (UCV) Caracas Venezuela; 5 Health Sciences Research Institute, Universidad Nacional de Asunción, San Lorenzo, Paraguay. Universidad Nacional de Asunción Health Sciences Research Institute Universidad Nacional de Asunción San Lorenzo Paraguay; 6 Biomedical Research Group, Faculty of Education and Science, Universidad de Sucre, Sincelejo, Colombia. Universidad de Sucre Biomedical Research Group Faculty of Education and Science Universidad de Sucre Sincelejo Colombia; 7 Research, Innovation and Development Division, Pyrogen S.A.S, Sucre, Colombia. Research, Innovation and Development Division, Pyrogen S.A.S, Sucre Colombia

**Keywords:** Leishmaniasis, Parasites, Didelphis, Colombia, DNA, Kinetoplast

## Abstract

We studied the prevalence of infection by trypanosomatid parasites in *Didelphis marsupialis* and its relationship with morphological/age aspects in a rural area of El Carmen de Bolivar, Colombia. Five visits were made to the Vereda El Alférez; each of which lasted three consecutive nights. During these visits, Tomahawk® traps were installed in the peridomestic and wild ecotopes of the Vereda El Alférez. Body measurements, sex and age were determined from the collected animals. Blood was extracted by cardiopuncture, after sedation, in order to obtain total deoxyribonucleic acid (DNA) and amplify the conserved region of the kinetoplast minicircle DNA (kDNA) of parasitic trypanosomatids. The association between morphological parameters of didelphids and their frequency of infection by parasitic trypanosomatids was determined by binomial regression. Thirty *D. marsupialis* specimens (60.0% females and 40.0% males/66.7% adults and 33.3% juveniles) were collected. Molecular diagnosis revealed a frequency of trypanosomatid parasite infection of 46.7%. Stage (p=0.024) was a determinant for infection. We discuss the role of *D. marsupialis* as a potential reservoir of parasitic trypanosomatids in the Vereda El Alférez.

## INTRODUCTION

Leishmaniasis is a neglected disease, and is very important for public health due to its high incidence and wide geographical distribution. According to the World Health Organization (WHO), leishmaniasis is found in 98 countries, with more than 12 million people infected with 20,000 to 30,000 deaths per year. More than 350 million people are estimated to be at risk of suffering from this type of zoonosis, with new cases ranging from 700,000 to one million, and mainly affecting populations living in areas with difficult access, lack of basic services, lack of education and low income ^1^.

In Colombia, more than 11 million people are estimated to be at risk of leishmaniasis, with 12,000 new cases reported annually since 2005 ^2^. During 2019, 5082 cases were reported, of which 98.9% corresponded to cutaneous leishmaniasis (CL), 1.0% to mucocutaneous leishmaniasis (MCL) and 0.2% to visceral leishmaniasis (VL) ^3^.

This zoonosis is caused by flagellates of the genus *Leishmania* (*Kinetoplastea*: *Trypanosomatidae*). The life cycle of these flagellates takes place in phlebotomine insects (*Diptera*: *Psychodidae*, *Phlebotominae*) that act as vectors; mammals of up to seven orders that act as reservoirs ^4^. Reports show that the frequency of *Leishmania* infection in rodents and marsupials is high, with *Didelphis marsupialis* (*Didelphimorphia*: *Didelphidae*) being the primary wild reservoir in several regions of Latin America, including Colombia ^4^^-^^7^. The distribution of leishmaniasis in humans coincides with the distribution of vectors and potential reservoirs within the habitats they occupy, forming ecological systems that favor transmission cycles ^4^.

The municipality of El Carmen de Bolívar, located in Los Montes de María, northern Colombia, is one of the most important foci of VL in the country and has reported 325 human cases of leishmaniasis. Between 2008 and 2019, 75 cases of VL were reported in this region, which added to the presence of *Lutzomyia evansi*, one of the primary vectors ^3^^,^^8^. Many cases of this zoonosis have been reported in Los Montes de María, however, there are few studies on wild reservoirs in this locality, mainly in the veredas, which are defined as rural nucleus close to an urban center with 50 to 1200 inhabitants. Therefore, the aim of this study was to evaluate the role of *D. marsupialis* as a potential reservoir of trypanosomatid parasites in this area of northern Colombia.

**KEY MESSAGES**

Motivation for the study. A leishmaniasis outbreak in Vereda El Alférez, El Carmen de Bolívar, in the Colombian Caribbean, provided an opportunity to determine the presence of trypanosomatid parasites in cardiac blood of marsupials and the relationship of this infection with their morphological characteristics and age.

Main findings. The frequency of infection with trypanosomatid parasites was of 46.7% (14/30) among the collected marsupials. The developmental stage was the determining variable for infection.

Implications. The synanthropic habits of *Didelphis marsupialis* and the frequency of infection in cardiac blood by trypanosomatid parasites suggest it plays a role as a potential reservoir of parasites causing Chagas disease or leishmaniasis, which represents a risk for the emergence of new foci of zoonosis in the region.

## THE STUDY

### Study area

**Figure 1:**
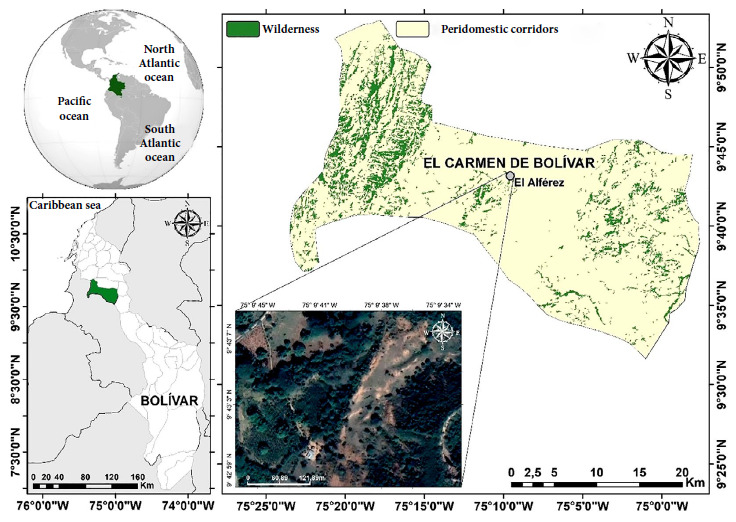
Map of the study area for the collection of *Didelphis marsupialis* in Vereda El Alférez, Colombia.

The research was conducted in Vereda El Alférez, a rural area of the municipality of El Carmen de Bolívar (Department of Bolívar, 09º45’38.0” N - 075º10’19.1” W), located 200 meters above sea level (masl) with a temperature between 26-30 °C, bimodal rainfall and relative humidity between 75-85% ^9^. Human settlements in this area are located in a tropical dry forest, in which the inhabitants grow fruit trees, mainly cocoa, avocado and timber trees, both sources of economic activity in the region (Figure 1).

### Collection and blood sampling of wild mammals

Five samplings were conducted from January 2018 to April 2019, using 21 Tomahawk® traps (66.04 cm × 22.86 cm × 22.86 cm) for three consecutive nights from 18:00 to 06:00 hours. Sampling was carried out in the peridomestic ecotope (PE), which corresponds to the surroundings of the dwelling (a diameter of up to 50 m), where domestic animals (poultry, pigs and cows) and synanthropic animals (rodents, marsupials and armadillos) can be found, as well as in the wild ecotope (S) (inside of the forest, 1 km from the PE) ^9^. Sampling both ecotopes required a total of 3024 hours.

The collected animals were taxonomically identified and then anesthetized for morphometric measurement, this included total length, tail length and leg length, as well as weight estimation; blood samples were also obtained following the procedure described by Ardila *et al*. ^8^. Each animal was implanted sub-dermally with a radio-frequency identification (RFID) device for tracking and recording of their eventual recapture. The animals were released after recovering from anesthesia. Blood samples were labeled and refrigerated at -20°C for molecular analysis at the Biomedical Laboratory of the Biomedical Research Group of the University of Sucre. All specimens were legally collected in compliance with the “Permiso Marco de Recolectamiento de Especímenes de Especies Silvestres de la Diversidad Biológica con Fines de Investigación Científica No Comercial”, Resolution 0391 of April 11, 2016 issued by the Autoridad Nacional de Licencias Ambientales (ANLA), granted to the Universidad de Sucre.

### Molecular detection of trypanosomatid parasites

The Wizard® Genomic DNA purification kit (Promega Corporation: Madison, Wisconsin, USA) was used for extracting total deoxyribonucleic acid (DNA) from blood samples, following the manufacturer’s instructions.

The NanoDrop 1000TM (Thermo Scientific, Massachusetts, USA) was used to quantify total DNA concentration. Molecular diagnostic was carried out by polymerase chain reaction (PCR) amplification of the highly repetitive sequence of kinetoplast minicircle DNA (kDNA) using primers 13A (5’-GGCCCACTATATTACACACCAACCAACCC-3’) and 13B (5’-GGGGGGTAGGGGGGCGTTCTCTGCGAA-3), which delimit a 120-bp fragment ^10^. The primers XAHR 17 (5’-CGGAACCGCTCCTCATTGCC-3’) and XAHR 20 (5’-ACCCACACTGTGCCCATCTA-3’) were used for the detection of the vertebrate characteristic β-actin gene as internal control, which delimit a 289-bp fragment ^11^. DNA from *Leishmania guyanensis* was used as positive control and DNA from an infection-free opossum was used as negative control.

The PCR mix had a final volume of 23.0 µL of which 3.0 µL corresponded to blank DNA and the remaining 20 µL to the reaction mix, which contained 2.4 µL of PCR buffer (5X), 1.25 µL of Taq DNA polymerase (Thermo SCIENTIFIC), 0.18 µL of primers 13A and 13B, 0.12 µL of primers XAHR 17 and XAHR 20 and 5.16 µL of sterile high molecular grade milliQ water.

PCR was carried out on the Veriti 96 Well thermal cycler (Applied Biosystems, Carlsbad, USA®) under denaturing conditions at 95.0°C for 20 sec (40 cycles), alignment at 57, 6°C for 40 sec (40 cycles) and extension at 72°C for 5 min. The PCR products (7 µL) were subjected to horizontal electrophoresis (80 v for 70 min) in 1.5% agarose gel developed with ethidium bromide prepared in TBE (Tris-Borate-EDTA) buffer 5X, and visualized in Quantum-STA photo documentation system (Bio-Rad, California, USA®).

Positive samples revealed the presence of 120 bp bands by using the positive controls and a 1000 bp molecular size marker (Fermentas, Thermo Fisher Scientific®, Massachusetts, USA) as reference. Additionally, strains from species of the genus *Trypanosoma*: *Trypanosoma cruzi* and *T. rangeli* were assessed. The Primer-Blast tool (available online at the National Center for Biotechnology Information [NCBI]) was used to conduct a local alignment search to evaluate the specificity of the primers.

### Data analysis

Descriptive statistics were used to analyze the abundance of mammals and the frequency of infection with trypanosomatid parasites. A binomial regression model (Statgraphics version 17) was used to assess the associations between the stage, sex and morphology of the collected mammals with the frequency of infection.

### Ethical aspects

The study was approved by the Ethics Committee of the Universidad del Atlántico, Puerto Colombia, Colombia, with the code 21-10.

## FINDINGS

**Figure 2:**
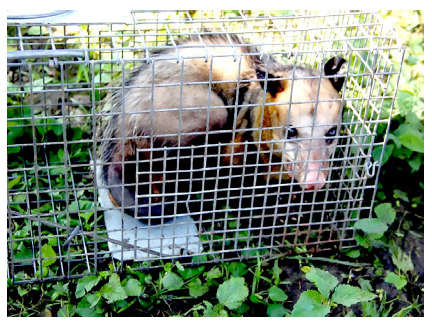
*Didelphis marsupialis* specimen in a Tomahawk® trap collected in the village of El Alférez, Municipality of El Carmen de Bolívar, Colombia.

Thirty specimens of *D. marsupialis* were collected (Figure 2), of which 66.7% (20/30) were adults and 33.3% (10/30) juveniles. Males accounted for 40.0% (12/30) and females for 60.0% (18/30), of which 33.3% (6/18) had pups in the marsupium at a rate of seven pups per female (Table 1).

**Table 1:** Morphological data of *Didelphis marsupialis* specimens collected in Vereda El Alférez, El Carmen de Bolívar, Colombia.

Specimen	Stage (Age)	Sex	Weight (g)	Body measurement (mm)				No. of cubs	PCR
				TL	TL	LL	EL				
1 (Dm001)	Adult	♂	1700	710	400	46	45	N/A	+
2 (Dm002)	Juvenile	♂	1384	825	375	41	48	N/A	+
3 (Dm003)	Juvenile	♂	1612	830	430	47	48	N/A	+
4 (Dm004)	Adult	♀	1400	850	450	47	46	7	+
5 (Dm005)	Adult	♀	1269	840	360	120	48	7	+
6 (Dm006)	Adult	♀	1270	860	390	50	41.9	6	-
7 (Dm007)	Adult	♀	1042	790	340	35	40	6	+
8 (Dm008)	Juvenile	♂	996	840	310	40	50	N/A	+
9 (Dm009)	Adult	♀	1719	910	430	50	60	7	-
10 (Dm010)	Adult	♂	2169	1010	450	160	70	N/A	+
11 (Dm011)	Adult	♂	2409	1000	410	36	46	N/A	+
12 (Dm012)	Adult	♂	2909	1020	380	128	41	N/A	+
13 (Dm015)	Juvenile	♂	1241	780	380	150	60	N/A	-
14 (Dm016)	Juvenile	♀	411	860	270	120	40	N/A	+
15 (Dm018)	Juvenile	♀	547	690	340	150	50	N/A	+
16 (Dm023)	Adult	♀	2020	910	440	180	50	7	+
17 (Dm024)	Juvenile	♀	353	610	310	120	30	N/A	-
18 (Dm025)	Juvenile	♀	157	650	300	130	40	N/A	-
19 (Dm026)	Adult	♂	1675	850	400	160	46	N/A	-
20 (Dm027)	Juvenile	♀	895	1080	360	120	35.3	N/A	+
21 (Dm028)	Adult	♀	781	1050	380	120	43.18	N/A	-
22 (Dm029)	Adult	♀	1172	1180	380	140	37.99	N/A	-
23 (Dm030)	Adult	♀	1052	1120	350	130	44.2	N/A	-
24 (Dm031)	Adult	♀	1256	1220	400	150	49.34	N/A	-
25 (Dm032)	Adult	♀	1055	1070	350	150	25.4	N/A	-
26 (Dm033)	Adult	♂	1790	1240	400	150	26,9	N/A	-
27 (Dm034)	Adult	♂	1975	840	340	115	35	N/A	-
28 (Dm035)	Adult	♀	2471	790	340	130	50	N/A	-
29 (Dm036)	Juvenile	♂	340	420	180	70	30	N/A	-
30 (Dm038)	Adult	♀	1600	1170	340	150	50	N/A	-

♀: female; ♂: male; g: grams; TL: total length; TL: tail length; LL: leg length; EL: ear length, mm: millimeters; +: positive sample; -: negative sample; N/A: not applicable.

On average, male specimens weighted more than females (1683g vs. 1138g), but females, on average, were longer than males (362mm vs. 244mm).

**Figure 3:**
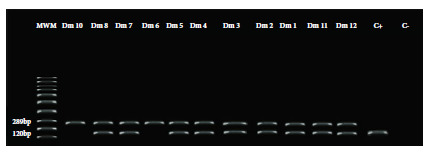
Agarose gel electrophoresis (1.5%-70 min/80 V) of 120 bp kinetoplast minicircle DNA (kDNA) reaction products from total blood of *Didelphis marsupialis* specimens, collected in rural area of the Municipality of El Carmen de Bolivar (Bolivar-Colombia). The 120 bp band corresponds to the amplified gene for the search of trypanosomatid parasites and the 289 bp band corresponds to the internal control of vertebrate DNA.

Molecular diagnosis of trypanosomatid parasites from the didelphids revealed an infection frequency of 46.7% (14/30), of which 57.0% (8/14) were adults and 43.0% (6/14) were juveniles (Figure 3). A 1:1 positivity ratio was found between males and females.

During the binomial regression analysis, we found that the only variable associated (p=0.024) with infection in individuals of *D. marsupialis* was the stage (8 adults and 6 juveniles), while the other variables such as sex (p=0.522), weight (p=0.131), total length (p=0.922), tail length (p=0.164) and leg length (p=0.074) showed values of p>0.05.

## DISCUSSION

We searched for trypanosomatids in *D. marsupialis*, a synanthropic mammal, typical of biological corridors between wild and urban ecotypes, in the Vereda del Alférez (El Carmen de Bolívar, Northern Colombia). We collected and assessed 30 specimens, of which 46.7% (14/30) were infected by trypanosomatids. These findings strengthen the study of epidemiological scenarios in one of the most important leishmaniasis hotspots and a hypoendemic area for American trypanosomiasis ^9^.

Vereda de El Alférez is located in a wooded area, with small households and structural deficiencies, which facilitates the creation of a humid and warm environment that favors the entry of tropical disease vectors ^9^^,^^12^^,^^13^. These conditions add to the domestic animal husbandry, which would favor contact between hematophagous insects, potential reservoir mammals and parasites ^13^.

*Didelphis marsupialis* is a very eclectic animal in terms of habitats and is considered to be one of the synanthropic mastofauna indicative of human environmental impact ^4^^,^^14^^,^^15^. In Latin America, this species has a high frequency of infection with leishmaniasis-causing agents ^4^. Particularly, in northern Colombia, these mammals have been found to be infected with *T. cruzi*, etiological agent of Chagas disease, with *T. rangeli*, which is infective for humans, but not pathogenic ^9^ and also with *Leishmania*^6^. In some cases, as in Venezuela, *D. marsupialis* may have a *Leishmania*/*T. cruzi* coinfection ^7^^,^^16^.

The ratio of three females for every two males of *D. marsupialis* found in the study area could be indicative of a low population size, given the possible existence of intra- and interspecific competition for resources and predation ^15^.

The weight of the specimens was similar to what was reported by Lozada *et al*. ^17^. The higher weight in males compared to females could be due to the reproductive period of females with higher energy expenditure, coinciding with lower water availability and higher solar irradiation during the sampling period (low precipitation) ^16^^,^^17^.

The only variable associated with the frequency of trypanosomatid parasite infection in the population of *D. marsupialis* was the developmental stage (57.0% positive adults and 43.0% positive juveniles), showing that adults are more likely to be infected than juveniles, probably because adult males with nocturnal and nomadic habits are exposed to the vectors for much longer than juveniles, as are females with cubs that remain in burrows in sympatry with the hematophagous insects ^5^^,^^8^.

In Colombia, studies related to the identification of trypanosomatid parasites in reservoirs mainly focus on domestic animals (mainly dogs) in constant direct interaction with humans and involved in the maintenance cycle of *Leishmania chagasi*^18^ and *T. cruzi*^19^. In Sincelejo, Sampués, and Oveja, municipalities in the Department of Sucre, northern Colombia, infection rates of 34.9%, 35.7%, and 11.1%, respectively, have been reported ^18^. No studies have been carried out in the study area searching for parasites of the genus *Leishmania* and *Trypanosoma* in canines.

Trypanosomatid parasites usually have macrophagotropism in “recruiter” tissues, therefore the fact that 46.7% of the specimens were positive for trypanosomatid parasites by cardiac blood PCR is unusual in leishmaniasis cases. This finding could be related to the presence of other trypanosomatid parasite species, such as *T. cruzi* and *T. rangeli* that have been reported in the area ^9^.

PCR results showed the amplification of the conserved region of the minicircle of the *Leishmania* species in the processed samples, however, a band of the same size also amplified in the strains of *T. cruzi* and *T. rangeli* evaluated later. Likewise, the results of the local alignment search carried out with Primer-Blast, showed that these primers also bind to such parasites, which contrasts with what is described in the literature for the specificity of this marker, thus broadening the pool of detectable organisms to other trypanosomatid parasites, but reducing the specificity of the test. The findings represent a risk factor for human populations since all these parasites are involved in the etiology of neglected tropical diseases transmitted by hematophagous insects, therefore, they should be taken into account for the design and evaluation of control strategies ^10^.

The infection of *D. marsupialis* with trypanosomatids shows a dynamic equilibrium in the study area, which ensures the survival of the parasites, not only in time, but also regarding the geography, due to its high population growth rate, ecological eclecticism ^15^, as well as its history as a reservoir in Colombia ^6^ and Venezuela ^7^^,^^16^.

A limitation of the study is that we did not characterize the species of circulating trypanosomatid parasites by using more specific genetic markers or sequencing. Limited funding contributed to this limitation.

In conclusion, the synanthropic behavior of these vertebrates, the frequency of infection with trypanosomatid parasites and the dynamic equilibrium between the parasite and the vertebrates imply the presence of rural cycles of zoonotic transmission that could eventually affect humans in Vereda El Alférez. Likewise, our results demonstrate the need for greater surveillance of vector-borne diseases, since these animals tend to be parasite amplifiers or transmission bridges between rural and urban cycles, representing an additional risk.
