# Supplementary material for: Molecular detection of the natural infection by trypanosomatid parasites in Didelphis marsupialis from a rural area in northern Colombia
Source: Rev Peru Med Exp Salud Publica. 2023 Mar 24;40(1):79–85. doi: 10.17843/rpmesp.2023.401.11573 (PMC10953644; doi:10.17843/rpmesp.2023.401.11573)
Supplement: Translation

# Translation: Detección molecular de la infección natural por parásitos tripanosomátidos en *Didelphis marsupialis* de una zona rural del norte de Colombia

**Authors:** Marlon M. Ardila, Yoselin Villadiego, Leidi Herrera, Wendy Zabala-Monterroza, Alveiro Pérez-Doria

**Affiliations:** 1 Facultad de Ciencias Básicas y Biomédicas, Universidad Simón Bolívar, Barranquilla, Colombia.; 2 Departamento de Ciencia Animal, Facultad de Ciencias Veterinarias, Universidad de Concepción, Chillán, Chile.; 3 Grupo Interdisciplinario en Ciencias Marinas y Ambientales (GICMARA), Facultad de Ciencias Básicas, Universidad del Atlántico, Puerto Colombia, Colombia.; 4 Centro de Ecología y Evolución, Instituto de Zoología y Ecología Tropical (IZET), Facultad de Ciencias, Universidad Central de Venezuela (UCV), Caracas, Venezuela.; 5 Instituto de Investigaciones en Ciencias de la Salud, Universidad Nacional de Asunción, San Lorenzo, Paraguay.; 6 Grupo Investigaciones Biomédicas, Facultad de Educación y Ciencias, Universidad de Sucre, Sincelejo, Colombia.; 7 División de Investigación, innovación y desarrollo, Pyrogen S.A.S, Sucre, Colombia.

**Keywords:** Leishmaniasis, Parásitos, Didelphis, Colombia, ADN de Cinetoplasto

## Abstract

Se evaluó la prevalencia de infección por parásitos tripanosomátidos en *Didelphis marsupialis* y su relación con los aspectos morfológicos/etarios en una zona rural de El Carmen de Bolívar, Colombia. En cinco visitas (2018-2019) se instalaron trampas Tomahawk® en los ecótopos peridoméstico y silvestre en la Vereda El Alférez, durante tres noches consecutivas/visita. A los animales recolectados, se les determinaron medidas corporales, sexo y edad; y se les extrajo sangre por cardiopuntura, previa sedación, para extracción del ácido desoxirribonucleico (ADN) total y amplificación de la región conservada del ADN de minicírculos de kinetoplasto (ADNk) de parásitos tripanosomátidos. La asociación entre parámetros morfológicos de los didélfidos y su frecuencia de infección por parásitos tripanosomátidos fue determinada mediante una regresión binomial*.* Se recolectaron 30 individuos de *D. marsupialis* (60,0% hembras y 40,0% machos/66,7% adultos y 33,3% juveniles). El diagnóstico molecular reveló una frecuencia de infección por parásitos tripanosomátidos del 46,7%. El estadio (p=0,024) fue determinante para la infección. Se discute el papel de *D. marsupialis* como potencial reservorio de parásitos tripanosomátidos en la zona evaluada.

## INTRODUCCIÓN

La leishmaniasis es una enfermedad desatendida de importancia en salud pública, debido a su alta incidencia y amplia distribución geográfica. Según la Organización Mundial de la Salud (OMS), la leishmaniasis se encuentra en 98 países, con más de 12 millones de personas infectadas con 20 000 a 30 000 muertes al año. Se estima que, más de 350 millones de personas se encuentran en riesgo de padecer esta zoonosis, con casos nuevos que oscilan entre 700 000 y un millón, afectando principalmente a las poblaciones que viven en zonas de difícil acceso, con ausencia de los servicios básicos, nivel educativo e ingresos económicos bajos ^1^.

En Colombia, se estima que más de 11 millones de personas se encuentran en riesgo de padecer leishmaniasis, reportándose 12 000 casos nuevos anuales desde el año 2005 ^2^. Para el año 2019, se presentaron 5082 casos, de los cuales el 98,9% correspondió a leishmaniasis cutánea (LC), 1,0% a leishmaniasis mucocutánea (LMC) y el 0,2% a leishmaniasis visceral (LV) ^3^.

Esta zoonosis es causada por flagelados del género *Leishmania* (Kinetoplastea: *Trypanosomatidae*), los cuales desarrollan su ciclo de vida entre insectos flebotomíneos (Diptera: *Psychodidae*, *Phlebotominae*) que actúan como vectores y mamíferos de hasta siete órdenes que ejercen como reservorios ^4^. Roedores y marsupiales, han sido reportados con elevada frecuencia de infección por *Leishmania,* siendo *Didelphis marsupialis* (Didelphimorphia: *Didelphidae*), el reservorio silvestre primario en varias regiones de Latinoamérica, incluyendo Colombia ^4^^-^^7^. La distribución de la leishmaniasis en humanos coincide con la distribución de los vectores y potenciales reservorios, enmarcados en los hábitats que ocupan, conformando sistemas ecológicos que favorecen los ciclos de transmisión ^4^.

En el Municipio de El Carmen de Bolívar, localizado en Los Montes de María, al norte de Colombia, uno de los focos más importantes de LV del país; se han registrado 325 casos de leishmaniasis en humanos. Entre 2008 y 2019, se registraron 75 casos de LV en esta región, lo cual se sumó a la presencia de *Lutzomyia evansi,* uno de los vectores primarios ^3^^,^^8^. Los Montes de María presenta muchos registros de esta zoonosis, sin embargo, son pocos los estudios sobre los reservorios silvestres en esta localidad, principalmente en las veredas, definidas como un núcleo rural cercano a un centro urbano con la presencia de 50 a 1200 habitantes. Por lo tanto, el objetivo de este estudio fue evaluar el papel de *D. marsupialis* como potencial reservorio de parásitos tripanosomátidos en esta área del norte de Colombia.

**MENSAJES CLAVE**

Motivación para realizar el estudio. La aparición de un foco de leishmaniasis en la Vereda El Alférez, El Carmen de Bolívar, del Caribe colombiano, motivó el interés por conocer la presencia de parásitos tripanosomátidos en sangre cardíaca de marsupiales y la relación de esta infección con sus caracteres morfológicos y grupos etarios.

Principales hallazgos. Los marsupiales recolectados presentaron una frecuencia de infección con parásitos tripanosomátidos del 46,7% (14/30). El estadio de desarrollo resultó la variable determinante para la infección.

Implicancias. Los hábitos sinantrópicos de *Didelphis marsupialis* y la frecuencia de infección en sangre cardíaca por parásitos tripanosomátidos sugieren su papel como un reservorio potencial de parásitos causantes de la enfermedad de Chagas o leishmaniasis, que representa un riesgo para el establecimiento de nuevos focos de zoonosis en la región.

## EL ESTUDIO

### Área de estudio

La investigación fue realizada en la Vereda El Alférez, zona rural del Municipio de El Carmen de Bolívar (Departamento de Bolívar, 09º45’38.0” N - 075º10’19.1” O), ubicado a 200 metros sobre el nivel del mar (msnm) con temperatura entre 26-30 °C, pluviosidad bimodal y humedad relativa entre 75-85% ^9^. Los asentamientos humanos en esta vereda están sobre un bosque seco tropical, en el cual, los pobladores desarrollan cultivos de árboles frutales con predominio del cacao, aguacate y árboles maderables, ambas fuentes de la actividad económica de la región (Figura 1).

**Figura 1:** Mapa del área de estudio para la recolecta de *Didelphis marsupialis* en la Vereda El Alférez, Colombia.

### Recolecta y toma de muestras de sangre en mamíferos silvestres

Se realizaron cinco muestreos desde enero de 2018 hasta abril de 2019, usando 21 trampas Tomahawk® (66,04 cm × 22,86 cm × 22,86 cm) por tres noches consecutivas desde las 18:00 a 06:00 horas en el ecótopo peridoméstico (PD), que corresponde a los alrededores de la vivienda (hasta 50 m en diámetro), con presencia de animales domésticos (aves de corral, cerdos y vacas) y referencia de animales sinantrópicos (roedores, marsupiales y armadillos), y en el ecótopo silvestre (S) (área interna al bosque a 1 km del PD) ^9^. Para el muestreo de ambos ecótopos se tuvo un esfuerzo de captura de 3024 horas.

Los individuos recolectados e identificados taxonómicamente fueron anestesiados para tomar sus medidas morfométricas, tales como longitud total, longitud de la cola y la longitud de la pata, así como para la estimación del peso y toma de muestras de sangre siguiendo el procedimiento descrito por Ardila *et al.*^8^. A cada animal le fue implantado por vía subdérmica un sistema por radiofrecuencia (Radio-frequency identification [RFID], por sus siglas en inglés), para su seguimiento y registro de eventual recaptura. Los animales fueron liberados, una vez recuperados de la anestesia, y las muestras de sangre fueron rotuladas y refrigeradas a -20 °C para su análisis molecular en el Laboratorio de Biomédicas del grupo Investigaciones Biomédicas de la Universidad de Sucre. Todos los individuos fueron legalmente recolectados de acuerdo con el «Permiso Marco de Recolección de Especímenes de Especies Silvestres de la Diversidad Biológica con Fines de Investigación Científica No Comercial», Resolución 0391 del 11 de abril de 2016 expedido por la Autoridad Nacional de Licencias Ambientales (ANLA), otorgado a la Universidad de Sucre.

### Detección molecular de parásitos tripanosomátidos

La extracción del ácido desoxirribonucleico (ADN) total de las muestras de sangre fue realizada con el kit de purificación de ADN de Wizard® Genomic (Promega Corporation: Madison, Wisconsin, USA), siguiendo las instrucciones del fabricante.

La concentración de ADN total fue cuantificada usando un NanoDrop 1000^TM^ (Thermo Scientific, Massachusetts, USA) y el diagnóstico molecular fue realizado mediante la amplificación por reacción en cadena de la polimerasa (PCR, por sus siglas en inglés) de la secuencia altamente repetitiva del ADN de los minicírculos del kinetoplasto (ADNk), usando los cebadores 13A (5’-GGCCCACTATATTACACCAACCC-3’) y 13B (5’-GGGGTAGGGGCGTTCTGCGAA-3), los cuales delimitan un fragmento de 120 pb ^10^. Los cebadores XAHR 17 (5’-CGGAACCGCTCATTGCC-3’) y XAHR 20 (5’-ACCCACACTGTGCCCATCTA-3’) fueron usados para la detección del gen *β-*actina característicos de vertebrados como control interno, los cuales delimitan un fragmento de 289 pb ^11^. Como control positivo se tomó el ADN de *Leishmania guyanensis* y como control negativo el ADN de una zarigüeya libre de la infección.

La mezcla de PCR fue realizada con un volumen final de 23,0 µL de los cuales 3,0 µL correspondieron al ADN blanco y los 20 µL restantes a la mezcla de reacción, la cual contuvo 2,4 µL de búfer PCR (5X); 1,25 µL de Taq ADN polimerasa (Thermo SCIENTIFIC); 0,18 µL de los cebadores 13A y 13B; 0,12 µL de los cebadores XAHR 17 y XAHR 20 y 5,16 µL de agua miliQ estéril de alto grado molecular.

La PCR fue llevada a cabo en el termociclador Veriti 96 Well (Applied Biosystems, Carlsbad, USA®) en condiciones de desnaturalización a 95,0 °C por 20 seg (40 ciclos), alineamiento a 57,6 °C por 40 segundos (40 ciclos) y extensión a 72 °C por 5 min Los productos de PCR obtenidos (7 µL) fueron sometidos a electroforesis horizontal (80 v por 70 min) en gel de agarosa al 1,5% revelado con bromuro de etidio preparado en búfer TBE (Tris-Borato-EDTA) 5X, y visualizados en sistema de foto documentación Quantum-STA (Bio-Rad, California, USA®).

Las muestras positivas revelaron presencia de bandas de 120 pb, utilizando como referente los controles positivos y un marcador de tamaño molecular 1000 pb (Fermentas, Thermo Fisher Scientific®, Massachusetts, USA). Adicionalmente, se evaluaron cepas de especies del género *Trypanosoma*: *Trypanosoma cruzi* y *T. rangeli*, y con la herramienta Primer-Blast (disponible en línea en el Centro Nacional de Información Biotecnológica [NCBI, por sus siglas en Inglés]) se realizó una búsqueda de alineamiento local para evaluar la especificidad de los cebadores usados.

### Análisis de los datos

La abundancia de mamíferos recolectados y la frecuencia de infección con parásitos tripanosomátidos fue analizada con estadística descriptiva. Se buscaron las asociaciones entre estadio, sexo y porte morfológico de los mamíferos recolectados con respecto a la frecuencia de infección mediante una regresión binomial, usando Statgraphics versión 17.

### Aspectos éticos

El estudio fue aprobado por el Comité de Ética de la Universidad del Atlántico, Puerto Colombia, Colombia, bajo el código de aprobación 21-10.

## HALLAZGOS

Se recolectaron 30 individuos de *D. marsupialis* (Figura 2), de los cuales el 66,7% (20/30) fueron adultos y el 33,3% (10/30) juveniles. El 40,0% (12/30) correspondió a machos y el 60,0% (18/30) a hembras, de las cuales el 33,3% (6/18) presentaron crías en el marsupio a razón de siete crías por hembra (Tabla 1).

**Figura 2:** Individuo de *Didelphis marsupialis* en trampa Tomahawk**®** recolectado en la vereda El Alférez, Municipio de El Carmen de Bolívar, Colombia.

**Tabla 1:** Datos morfológicos de individuos de *Didelphis marsupialis* recolectados en la Vereda El Alférez, El Carmen de Bolívar, Colombia.

Individuo	Estadio (edad)	Sexo	Peso (g)	Medidas corporales (mm)				No. de crías	Análisis PCR
				LT	LC	LP	LO		
1 (Dm001)	Adulto	♂	1700	710	400	46	45	N/A	+
2 (Dm002)	Juvenil	♂	1384	825	375	41	48	N/A	+
3 (Dm003)	Juvenil	♂	1612	830	430	47	48	N/A	+
4 (Dm004)	Adulto	♀	1400	850	450	47	46	7	+
5 (Dm005)	Adulto	♀	1269	840	360	120	48	7	+
6 (Dm006)	Adulto	♀	1270	860	390	50	41,9	6	-
7 (Dm007)	Adulto	♀	1042	790	340	35	40	6	+
8 (Dm008)	Juvenil	♂	996	840	310	40	50	N/A	+
9 (Dm009)	Adulto	♀	1719	910	430	50	60	7	-
10 (Dm010)	Adulto	♂	2169	1010	450	160	70	N/A	+
11 (Dm011)	Adulto	♂	2409	1000	410	36	46	N/A	+
12 (Dm012)	Adulto	♂	2909	1020	380	128	41	N/A	+
13 (Dm015)	Juvenil	♂	1241	780	380	150	60	N/A	-
14 (Dm016)	Juvenil	♀	411	860	270	120	40	N/A	+
15 (Dm018)	Juvenil	♀	547	690	340	150	50	N/A	+
16 (Dm023)	Adulto	♀	2020	910	440	180	50	7	+
17 (Dm024)	Juvenil	♀	353	610	310	120	30	N/A	-
18 (Dm025)	Juvenil	♀	157	650	300	130	40	N/A	-
19 (Dm026)	Adulto	♂	1675	850	400	160	46	N/A	-
20 (Dm027)	Juvenil	♀	895	1080	360	120	35,3	N/A	+
21 (Dm028)	Adulto	♀	781	1050	380	120	43,18	N/A	-
22 (Dm029)	Adulto	♀	1172	1180	380	140	37,99	N/A	-
23 (Dm030)	Adulto	♀	1052	1120	350	130	44,2	N/A	-
24 (Dm031)	Adulto	♀	1256	1220	400	150	49,34	N/A	-
25 (Dm032)	Adulto	♀	1055	1070	350	150	25,4	N/A	-
26 (Dm033)	Adulto	♂	1790	1240	400	150	26,9	N/A	-
27 (Dm034)	Adulto	♂	1975	840	340	115	35	N/A	-
28 (Dm035)	Adulto	♀	2471	790	340	130	50	N/A	-
29 (Dm036)	Juvenil	♂	340	420	180	70	30	N/A	-
30 (Dm038)	Adulto	♀	1600	1170	340	150	50	N/A	-

♀: hembra; ♂: macho; g: gramos; LT: longitud total; LC: longitud de la cola; LP: longitud de las patas; LO: longitud de las orejas, mm: milímetros; +: muestra positiva; -: muestra negativa; N/A: no aplica.

El peso promedio de los individuos fue relativamente mayor en machos con respecto al de las hembras (1683 g *vs.*1138 g), mientras que la longitud total fue relativamente mayor en hembras en relación con los machos (362 mm *vs.* 244 mm).

El diagnóstico molecular de los parásitos tripanosomátidos sobre los didélfidos recolectados, reveló, una frecuencia de infección del 46,7% (14/30), de los cuales el 57,0% (8/14) fueron adultos y el 43,0% (6/14) juveniles (Figura 3). Teniendo en cuenta el sexo, se encontró una proporción 1:1 para las muestras positivas.

**Figura 3:** Electroforesis en gel de agarosa (1,5%-70 min/80 V) de los productos de reacción de ADN de minicírculos de kinetoplasto (ADNk) de 120 pb, a partir de la sangre total de individuos de *Didelphis marsupialis*, recolectados en zona rural del Municipio de El Carmen de Bolívar (Bolívar-Colombia). La banda de 120 pb corresponde al gen amplificado para la búsqueda de parásitos tripanosomátidos y la banda de 289 pb corresponde al control interno de ADN de vertebrados.

En el análisis de regresión binomial, se encontró que la única variable asociada (p=0,024) a la infección en individuos de *D. marsupialis* fue el estadio (8 adultos y 6 juveniles), mientras que las otras variables tales como el sexo (p=0,522), peso (p=0,131), longitud total (p=0,922), longitud de la cola (p=0,164) y longitud de la pata (p=0,074); presentaron valores de p>0,05. 

## DISCUSIÓN

La presente investigación responde a la búsqueda de tripanosomátidos en *D. marsupialis*, mamífero sinantrópico, propio de corredores biológicos entre ecótopos silvestres y urbanos, en la Vereda del Alférez (El Carmen de Bolívar, Norte de Colombia) donde se registraron 30 individuos, de los cuales el 46,7% (14/30) presentó infección por estos hemoflagelados. Los hallazgos fortalecen el estudio de los escenarios epidemiológicos en uno de los focos más importantes para la leishmaniasis y zona hipoendémica para la tripanosomiasis americana ^9^.

La Vereda de El Alférez se localiza en una zona boscosa, con viviendas pequeñas y con deficiencias estructurales, lo cual facilita la creación de un ambiente húmedo y cálido que favorece la entrada de los vectores de enfermedades tropicales ^9^^,^^12^^,^^13^. Estas condiciones se suman al manejo del PD para la cría de animales domésticos, lo que favorecería el contacto entre insectos hematófagos, mamíferos reservorios potenciales y parásitos ^13^.

*Didelphis marsupialis* es un animal muy ecléctico en cuanto a los hábitats y se le considera dentro de la mastofauna sinantrópica indicadora de afectación ambiental humana ^4^^,^^14^^,^^15^. Esta especie, en Latinoamérica, tiene una elevada frecuencia de infección con agentes causantes de leishmaniasis ^4^. Específicamente, en el norte de Colombia se han encontrado infectados con *T. cruzi,* agente etiológico de la enfermedad de Chagas, con *T. rangeli,* que es infectivo para el humano, pero no patógeno ^9^ y también con *Leishmania*^6^*.* En algunos casos, como en Venezuela, *D. marsupialis* mantiene una coinfección *Leishmania/T. cruzi*^7^^,^^16^.

La proporción de tres hembras por cada dos machos de individuos de *D. marsupialis* hallados en el área de estudio podría ser indicativo de un tamaño poblacional bajo, ante una posible existencia de competencia a nivel intra e interespecífica por los recursos y depredación ^15^.

Los pesos hallados en los individuos, resultaron ser similares a los reportados por Lozada *et al.*^17^. El peso mayor en machos con respecto a las hembras podría deberse al periodo de reproducción de éstas con mayor gasto energético, coincidente con menor disponibilidad de agua y mayor irradiación solar en el período de muestreo (baja precipitación) ^16^^,^^17^.

La única variable asociada a la frecuencia de infección por parásitos tripanosomátidos en la población de *D. marsupialis* evaluada, fue el estadio del desarrollo (57,0% adultos positivos y 43,0% juveniles positivos); revelando que los adultos tienen mayor probabilidad de estar infectados en relación a los juveniles, probablemente porque los machos adultos de hábitos nocturnos y nómadas se exponen mucho más tiempo a los vectores que los juveniles, al igual que las hembras con crías que permanecen en las madrigueras en simpatría con los insectos hematófagos ^5^^,^^8^.

En Colombia, los estudios relacionados con la identificación de parásitos tripanosomátidos en reservorios están mayormente referidos a animales domésticos (principalmente el perro) en constante relación directa con el hombre e involucrados en el ciclo de mantenimiento de *Leishmania chagasi*^18^^)^ y *T. cruzi*^19^. En Sincelejo, Sampués y Oveja; municipios del Departamento de Sucre, al norte de Colombia, se han reportado tazas de infección de 34,9%, 35,7% y 11,1%; respectivamente ^18^. Para la vereda objeto de estudio no se han realizado trabajos encaminados a la búsqueda de parásitos del género *Leishmania* y *Trypanosoma* en caninos.

El hecho de que el 46,7% de los individuos estudiados estuvieron positivos a parásitos tripanosomátidos por PCR de sangre obtenida del corazón, es poco común en la leishmaniasis, en la cual el parásito tiene un macrofagotropismo en tejidos **«**reclutadores». Esta presencia en sangre podría corresponder a la presencia de otras especies de parásitos tripanosomátidos, como *T. cruzi* y *T. rangeli* que han sido informadas en el área ^9^.

Los resultados de PCR aplicada mostraron la amplificación de la región conservada del minicírculo de las especies del género *Leishmania* en la muestras procesadas, sin embargo, una banda del mismo tamaño también amplificó en las cepas de *T. cruzi* y *T. rangeli* evaluadas posteriormente, así mismo, los resultados de la búsqueda de alineamiento local realizada con Primer-Blast, mostró que estos cebadores también se unen a tales parásitos, lo que contrasta con lo descrito en la literatura para la especificidad de este marcador, de este modo se amplía la base de organismos detectables a otros parásitos tripanosomátidos, pero se reduce la especificidad de la prueba. Los hallazgos representan un factor de riesgo para las poblaciones humanas puesto que todos estos parásitos están involucrados en la etiología de enfermedades tropicales desatendidas transmitidas por insectos hematófagos, por lo tanto, se deben tener en cuenta para el diseño y la evaluación de estrategias de control ^10^.

La infección de *D. marsupialis* con tripanosomátidos alcanza un equilibrio dinámico en la zona lo cual asegura el mantenimiento de los parásitos, no solo en tiempo, si no también en la geografía, debido a su alta tasa de crecimiento poblacional, el eclecticismo ecológico ^15^ y sus antecedentes como reservorio en Colombia ^6^ y Venezuela ^7^^,^^16^.

El estudio necesitaría profundizar en la caracterización de la especie de parásito tripanosomátido circulante en esta zona, utilizando marcadores genéticos más específicos o alternativamente ir a la secuenciación, lo cual fue una limitante, en las condiciones de financiamiento de las que se disponía.

En conclusión, el comportamiento sinantrópico de estos vertebrados, la frecuencia de infección con parásitos tripanosomátidos y el equilibro dinámico observado entre el parásito y los vertebrados, indican la presencia de ciclos rurales de transmisión zoonótica lo cual eventualmente podrían afectar a los humanos de la Vereda El Alférez. Así mismo, evidencian la necesidad de mayor vigilancia de enfermedades transmitidas por vectores, ya que estos animales tienden a ser amplificadores de los parásitos o puentes de transmisión entre los ciclos rurales y urbano, constituyendo un elemento de riesgo adicional.
